# Prevalence of Back and Neck Pain Among Orthopedic Surgeons in Riyadh, Saudi Arabia

**DOI:** 10.7759/cureus.73535

**Published:** 2024-11-12

**Authors:** Abdulmajeed A Alzakri, Habib Ullah Chaudhary, Omar A Ababtain, Mohammed A Alshwieer, Nasser F AlSunbul

**Affiliations:** 1 Department of Orthopedics, College of Medicine, King Saud University, Riyadh, SAU; 2 Spine Surgery Unit, King Saud University Medical City, King Saud University, Riyadh, SAU; 3 Department of Orthopedics, King Faisal Specialist Hospital &amp; Research Center, Riyadh, SAU; 4 Department of Trauma Surgery, King Saud Medical City (KSMC), Riyadh, SAU; 5 Department of Orthopedic Surgery, Security Forces Hospital, Riyadh, SAU

**Keywords:** back pain, musculoskeletal disorders, neck pain, occupational health, orthopedic surgeons, saudi arabia

## Abstract

Background and objectives: Orthopedic surgeons' demanding work may negatively affect their health. This study examines the prevalence of musculoskeletal (MSK) issues, specifically back and neck pain, among orthopedic surgeons in Riyadh, Saudi Arabia, and explores contributing sociodemographic factors.

Materials and methods: We conducted an observational study that assessed the prevalence of back and neck pain among certified orthopedic surgeons using an online survey, which included Logistic regression for risk factors, one-way ANOVA for disability-contributing factors, and Tukey’s post-hoc test for subgroup analysis. Additionally, the EQ5D-Index and EQ5D-VAS scores were compared between those with and without back and neck pain using an unpaired t-test, with all tests maintaining a significance level of p<0.05.

Results: Neck pain was prevalent (58.1%; N=36) among the surgeons, primarily during their attending years, with severity significantly impacting medical caregiving. Age was a risk factor, with those aged 35-44 and over 55 years being at higher risk. Back pain was documented in 74.2% (N=46) of the cases, with severity influencing medical care seeking. Fatigue, decreased quality of life, practice adjustments, theater changes, absenteeism, and early retirement have been reported consequences of neck and back pain.

Conclusions: High rates of neck and back pain among Riyadh orthopedic surgeons, largely due to poor posture, were found. Age significantly influenced neck pain development. The study recommends posture and workplace ergonomic interventions to mitigate musculoskeletal pain's impact on surgeons' personal and professional lives.

## Introduction

Occupational musculoskeletal (MSK) disorders pose a serious health threat across the globe, representing up to 30% of work-related injuries in the United States [1]. However, the under-reported true prevalence is believed to be much higher, with an estimation that 7.0 to 10.5 million workers are afflicted with an occupational injury or illness annually [1]. Studies from Saudi Arabia indicate even higher prevalence rates of musculoskeletal disorders among medical staff, ranging from 51% to as high as 88.9% [2].

The Occupational Safety and Health Administration (OSHA) considers an injury or illness work-related if a work environment exposure or event was the direct cause or contributing factor or made a pre-existing condition notably worse [3]. Healthcare workers, particularly orthopedic surgeons, face an elevated risk of debilitating musculoskeletal injuries due to the physically demanding nature of their occupations [3].

The toll of work-related injuries among healthcare personnel amounts to an estimated $13.1 billion in costs and 2 million lost workdays annually [4]. In fact, up to two-thirds of orthopedic surgeons report experiencing at least one work-related musculoskeletal condition during their careers, with as many as one-third requiring surgery or time off work to address occupational injuries [3,5].

Lower back and neck pain are widely acknowledged as two of the most prevalent MSK injuries affecting orthopedic surgeons, according to a growing body of research. In Saudi Arabia, MSK disorders and the associated pain represent a significant occupational hazard for orthopedic surgeons, with previous studies indicating alarmingly high prevalence rates of lower back pain ranging from 23% to nearly 90%, alongside neck pain that varies from 14% to 74% globally [5,6]. In 2020, a study in Saudi Arabia revealed a high prevalence of lower back and neck pain (74.0% and 58.2%) among orthopedic surgeons [7]. Similarly, another study was carried out in the eastern region of Saudi Arabia aimed to investigate the prevalence among orthopedic residency surgeons reported the highest prevalence of lower back pain at 46% followed by neck pain at 39.7% with the youngest residents (R1s) exhibiting the highest rates compared to other orthopedic surgeons [8].

Numerous factors contribute to this elevated risk, such as extended operating room hours, prolonged standing with improper posture, and physically demanding tasks [3,6]. While previous studies indicate high rates of musculoskeletal conditions among surgeons globally and locally, more research is needed to fully understand the issue's magnitude and identify potential mitigating factors. Significant gaps remain regarding the prevalence of back and neck pain, specifically among Saudi orthopedic surgeons, including differences across demographic groups. Addressing this occupational health challenge is critical to alleviating the pain and suffering experienced by overburdened surgeons while sustaining their ability to provide high-quality orthopedic care now and in the future.

Our study was designed to investigate the prevalence of back and neck pain in Riyadh among orthopedic surgeons while exploring relationships between demographic factors including gender, age, and career seniority. By quantifying the effects on surgeon work performance and quality of life, this study aims to provide insights into preventive strategies and management for orthopedic surgeons affected by musculoskeletal pain.

## Materials and methods

This observational, cross-sectional study was conducted in Riyadh, Saudi Arabia, utilizing an online survey previously employed by Lucasti et al. [9]. The survey, comprising 37 questions (see Appendices), was distributed via email with modifications only in the units, which were converted to the International System of Units. Participants were provided with the period from June to November 2023 to complete the survey utilizing Google Forms. The survey aimed to ascertain the prevalence of back and neck pain among orthopedic surgeons in Riyadh and sought consent from each participant to use their data for this purpose.

Lucasti et al. reported a 77% prevalence of back and neck pain in western New York [9]. Based on these findings, we utilized a single proportion formula to calculate an appropriate sample size of 273, targeting a 95% confidence interval and an absolute precision of 0.05. Despite this calculation, we surveyed 300 orthopedic surgeons to accommodate a potential non-response rate of 10%.

The study population included all certified orthopedic surgeons, both fellows and consultants, practicing for at least one year in Riyadh City, Saudi Arabia. Exclusion criteria included surgeons with congenital deformities or a history of trauma that might impact their musculoskeletal function.

The survey collected demographic information such as age, sex, smoking history, and body measurements. It also inquired about professional details, including the level of training, specialty, years of practice, number of days spent operating per week, daily operating hours, and hours spent standing. Additionally, it explored the occurrence of work-related back and neck pains.

Statistical analysis

Data analysis was conducted using SPSS Statistics for Mac version 29.0 (IBM Corp., Armonk, NY, USA). We performed logistic regression to analyze back and neck pain risk factors. A one-way analysis of variance (ANOVA) was used to evaluate the characteristics and impact of these pains on disability (ODI scores). Significant findings from the ANOVA were further explored through Tukey’s post-hoc test to assess differences among subgroups and their effects on ODI scores. The EQ5D-Index and EQ5D-VAS scores for individuals with and without back and neck pain were compared using an unpaired t-test, with significance set at p<0.05.

Ethics and disclosure

The authors have no conflicts of interest, and the work has not received support or funding from a drug company. Informed consent was obtained from all participants to participate in the study. Research has been performed in accordance with the Declaration of Helsinki and approved by the Standing Committee for Scientific Research Committee, Deanship of Scientific Research, King Saud University, Riyadh, Saudi Arabia (IRB Approval No. KSU-HE-23-883).

## Results

Sixty-three individuals participated in the survey, although one chose not to share the results, leaving 62 valid responses for analysis. The majority (93.4%; N=58) were male surgeons. The age distribution of the participants revealed that the majority (58.8%; N=37 individuals) were younger than 44 years. Of these, 28.6% (N=18 individuals) were under 35 years old, and 30.2% (N=19 individuals) were aged between 35 and 44 years. A small proportion of participants, 9.5% (N=7), were aged 55 years or older (Table 1).

**Table 1:** Distribution of socio-demographic characteristics of study subjects. SD = Standard deviation.

Orthopedic surgeons (N=62)	Value
Age in years, n (%)	
<35	18 (28.6)
35 to 44	19 (30.2)
45 to 55	18 (28.6)
More than 55	7 (9.5)
Mean (±SD)	41.35 (±9.7)
Gender, n (%)	
Male	58 (93.4)
Female	4 (6.6)
Height, n (%)	
Less than 167.5 cm	14 (22.2)
170 to 185.5 cm	44 (69.8)
More than 188 cm	4 (6.3)
Mean (±SD)	175.1 (±9.1)
Weight, n (%)	
45 to 67.5 kg	6 (9.7)
68 to 90 kg	36 (58.1)
90.5 - 113 kg	16 (25.8)
>113.5 kg	4 (6.5)
Mean (±SD)	85.3 (±17.0)

The average height and weight of the participants were approximately 175.1 cm (68.93 inches) and 85.3 kg (188.05 pounds), respectively, in which the average BMI for participants was 27.8 kg/m2. There are 22 individuals (36.1%) reported having consumed tobacco products, 13 (21.3%) were current smokers, and 9 (14.8%) were former smokers. Mean hours of physical activity or exercise per week was 4.6 (±7.3), and 18.3% (11 study subjects) stated they do not engage in any kind of exercise on a weekly basis.

In terms of professional status, 65.5% (N=40 individuals) were attending physicians, and 34.4% (N=21 individuals) were fellows. Among the attending physicians, 12.1% (N=8 individuals) specialized in spine surgery. The remaining attending physicians were spread across various specialties, with pediatrics comprising 21.3%, spine at 12.1%, and both upper extremities and "others" specialties, each accounting for 6.6% (Table 2). One participant did not answer this question.

**Table 2:** Subspecialty distribution among attending physicians in the study.

Subspeciality	Respondents	Percentage
Spine	8	12.1%
Joint arthroplasty	3	4.9%
Trauma	3	4.9%
Foot and ankle	2	3.3%
Upper extremity	4	6.6%
Oncology	2	3.3%
Pediatrics	13	21.3%
Others	4	6.6%
Did not answer	1	2.44%
Total	40	100.00%

This study revealed a 58.1% (N= 36 individuals) prevalence of neck pain among orthopedic surgeons at some point in their careers (Table 3). Most of these participants indicated that their neck pain began while they were attending; 10 reported that the pain started during residency, and two reported experiencing pain prior to residency. Among those experiencing neck pain, 13.9% (N=five individuals) reported experiencing pain for more than four days per week, with 80% (N=4 of these five individuals) seeking medical attention. Meanwhile, 52.3% (N=19) experienced neck pain less than one day per week, with 31.6% (N=6) of these individuals seeking medical care. In terms of pain severity, 38.9% (N= 14 individuals) rated their pain as moderate to severe. Most participants (77.8%; N=28) reported experiencing neck pain for less than five years.

**Table 3:** Univariate and multivariate analysis of neck pain risk factors for healthcare seeking. CI = Confidence interval.

Neck pain characteristic	Prevalence of neck pain (n, %)	Sought medical care (n, %)	Univariate analysis		Multivariate analysis	
			Relative risk (95% CI)	P-value	Relative risk (95% CI)	P-value
Frequency per week, n (%)						
Less than 1 day	19 (52.3%)	6 (31.6%)	1		1	
1 to 3 days	12 (33.3%)	2 (16.7%)	0.43 (0.07 - 2.62)	0.363	0.14 (0.01 - 1.76)	0.129
More than 4 days	5 (13.9%)	4 (80.0%)	8.67 (0.79 - 95.09)	0.077	0.74 (0.03 - 18.11)	0.853
Duration of neck pain, n (%)						
Less than 5 years ago	28 (77.8%)	9 (32.1%)	1		1	
5 to 10 years ago	6 (16.7%)	2 (33.3%)	1.06 (0.16 - 6.88)	0.955	0.78 (0.09 - 6.91)	0.816
More than 10 years ago	3 (8.4%)	2 (66.7%)	4.22 (0.34 - 52.90)	0.264	3.28 (0.15 - 69.82)	0.447
Intensity of pain, n (%)						
Minimal to mild	22 (61.1%)	3 (13.6%)	1			
Moderate to severe	14 (38.9%)	9 (64.3%)	11.40 (2.22 - 58.56)	0.004	22.80 (1.95 - 266.07)	0.013
Overall (N=62)	36 (58.1%)	12 (33.3%)				

This study found that neck pain severity was the only statistically significant factor in the decision to seek medical care among those experiencing neck pain (p<0.05), with a relative risk of 22.8 (2.00, 266.1). Additionally, age was found to be the only statistically significant factor for developing neck pain, specifically for those between 35-44 years old and those above 55 years old, with relative risks of 10.3 and 15.8,(1.1, 93.2) and (1.1, 242.8), respectively, compared to those younger than 35 years old (p<0.05) (Tables 3, 4).

**Table 4:** Associations between demographic factors and neck pain prevelance in orthopedic surgeons. CI = Confidence interval.

Risk factor	Prevalence of neck pain (%)	Univariate analysis		Multivariate analysis	
		Relative risk (95% CI)	P-value	Relative risk (95% CI)	P-value
Age in years, n (%)					
<35	33.3	1		1	
35 to 44	73.7	5.60 (1.36 - 23.10)	0.017	10.29 (1.14 - 93.18)	0.038
45 to 55	61.1	3.14 (0.80 - 12.29)	0.100	5.92 (0.62 - 57.10)	0.124
More than 55	71.4	5.00 (0.74 - 33.78)	0.099	15.84 (1.03 - 242.78)	0.047
Gender, n (%)					
Male	58.6	1		1	
Female	50.0	0.71 (0.09 - 5.36)	0.740	1.08 (0.12 - 9.95)	0.948
Level of training, n (%)					
Resident/fellow	42.9	1		1	
Attending	65.9	2.57 (0.87 - 7.56)	0.086	1.74 (0.47 - 6.43)	0.406
Exercise hours per week, n (%)					
0	80.0	1		1	
1 to 3	54.5	0.30 (0.05 - 1.75)	0.181	0.25 (0.04 - 1.66)	0.152
4 to 6	47.6	0.23 (0.04 - 1.34)	0.101	0.25 (0.04 - 1.60)	0.143
More than 6	66.7	0.50 (0.06 - 4.00)	0.513	0.54 (0.06 - 4.90)	0.540
Smoking status, n (%)					
Smoker	46.2	1		1	
Non-smoker	64.1	2.08 (0.58 - 7.43)	0.258	1.30 (0.25 - 6.75)	0.752
Past smoker	50.0	1.17 (0.22 - 6.08)	0.855	0.70 (0.10 - 5.06)	0.721

**Figure 1:**
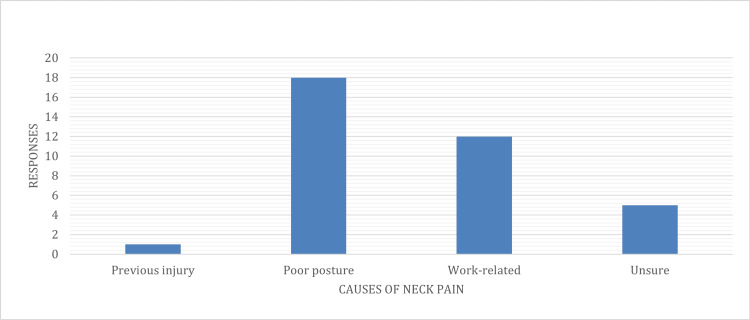
Subjects’ attribution of primary cause of the neck pain.

When asked about the cause of neck pain, 50% (N=18) of the surgeons attributed it to poor posture, 33.3% (N=12) believed it was work-related, 13.3% (N=5) were unsure, and 2.8% (N=1) attributed it to a previous injury (Figure 1).

This study reported a back pain prevalence of 74.2% ( N=47) among orthopedic surgeons (Table 5). Among these respondents, 20 (45.5%) stated that the onset of pain began during residency, 18 (40.9%) started experiencing pain, and six (13.6%) reported that pain began prior to residency. In terms of the frequency of pain, 47.8% (N=30) of the participants reported experiencing back pain less than one day per week, whereas 13% (N=6) reported experiencing pain four or more days per week. Additionally, 37% (N=23) of the participants indicated that they had experienced back pain one to three days per week. When asked to rank the severity of their pain, most participants (63%; N=40) classified their pain as minimal to mild, whereas 16 (34.8%) categorized their pain as moderate to severe. Of the 16 surgeons who reported experiencing moderate to severe pain, 10 sought medical care for back pain.

**Table 5:** Univariate and multivariate analysis of back pain risk factors for healthcare seeking. CI = Confidence interval.

Back pain characteristic	Prevalence of back pain (n, %)	Sought medical care (n, %)	Univariate analysis		Multivariate analysis	
			Relative risk (95% CI)	P-value	Relative risk (95% CI)	P-value
Frequency per week, n (%)						
Less than 1 day	22 (47.8%)	5 (22.7%)	1		1	
1 to 3 days	17 (37.0%)	6 (35.3%)	1.45 (0.37 - 5.71)	0.591	1.10 (0.20 - 5.87)	0.916
More than 4 days	6 (13.0%)	4 (66.6%)	3.56 (0.61 - 20.81)	0.159	1.79 (0.21 - 15.57)	0.596
Duration of back pain, n (%)						
Less than 5 years ago	25 (54.3%)	5 (20.0%)	1		1	
5 to 10 years ago	11 (24.0%)	4 (36.4%)	1.81 (0.39 - 8.39)	0.448	3.60 (0.55 - 23.51)	0.181
More than 10 years ago	11 (23.9%)	6 (54.5%)	3.80 (0.85 - 17.04)	0.081	5.27 (0.87 - 31.84)	0.070
Intensity of pain, n (%)						
Minimal to mild	29 (63.0%)	6 (20.7%)	1		1	
Moderate to severe	16 (34.8%)	10 (62.5%)	6.39 (1.65 - 24.73)	0.007	7.56 (1.54 - 37.18)	0.013
Overall (N=62)	46 (74.2%)	16 (34.8%)				

Analyses showed that only the severity of pain was statistically significant (p-value<0.05) in influencing those with back pain to seek medical care (Table 5). No factors were found to be statistically significant in developing back pain among orthopedic surgeons (Table 6). Moreover, the analyses demonstrated that only pain severity was statistically significant (p<0.05) in influencing patients with back pain to seek medical care (Table 5). No factors were found to be statistically significant in the development of back pain among orthopedic surgeons (Table 6).

**Table 6:** Associations between demographic factors and back pain in orthopedic surgeons. CI = Confidence interval. NA = Not applicable.

Risk factor	Prevalence of back pain (%)	Univariate analysis		Multivariate analysis	
		Relative risk (95% CI)	P-value	Relative risk (95% CI)	P-value
Age in years, n (%)					
<35	72.2	1		1	
35 to 44	80.0	1.44 (0.32 - 6.53)	0.635	1.14 (0.14 - 9.37)	0.906
45 to 55	61.1	0.60 (0.15 - 2.45)	0.481	0.38 (0.04 - 3.65)	0.404
More than 55	85.7	2.31 (0.22 - 24.32)	0.486	3.25 (0.17 - 60.70)	0.431
Gender, n (%)					
Male	72.4	1		1	
Female	75.0	1.05 (0.10 - 10.85)	0.970	1.30 (0.10 - 16.36)	0.840
Level of training, n (%)					
Resident/fellow	66.7	1		1	
Attending	75.6	1.24 (0.38 - 4.06)	0.722	2.22 (0.50 - 9.97)	0.296
Exercise hours per week, n (%)					
0	80.0	1		1	
1 to 3	77.3	0.54 (0.09 - 3.21)	0.494	0.50 (0.08 - 3.17)	0.458
4 to 6	61.9	0.63 (0.10 - 3.84)	0.612	0.67 (0.10 - 4.34)	0.671
More than 6	77.8	0.88 (0.10 - 7.95)	0.906	1.18 (0.11 - 12.32)	0.889
Smoking status, n (%)					
Smoker	77.0	1		1	
Non-smoker	74.4	0.87 (0.20 - 3.81)	0.853	0.80 (0.13 - 5.18)	0.818
Past smoker	60.0	0.45 (0.07 - 2.74)	0.386	0.42 (0.05 - 3.54)	0.424

**Figure 2:**
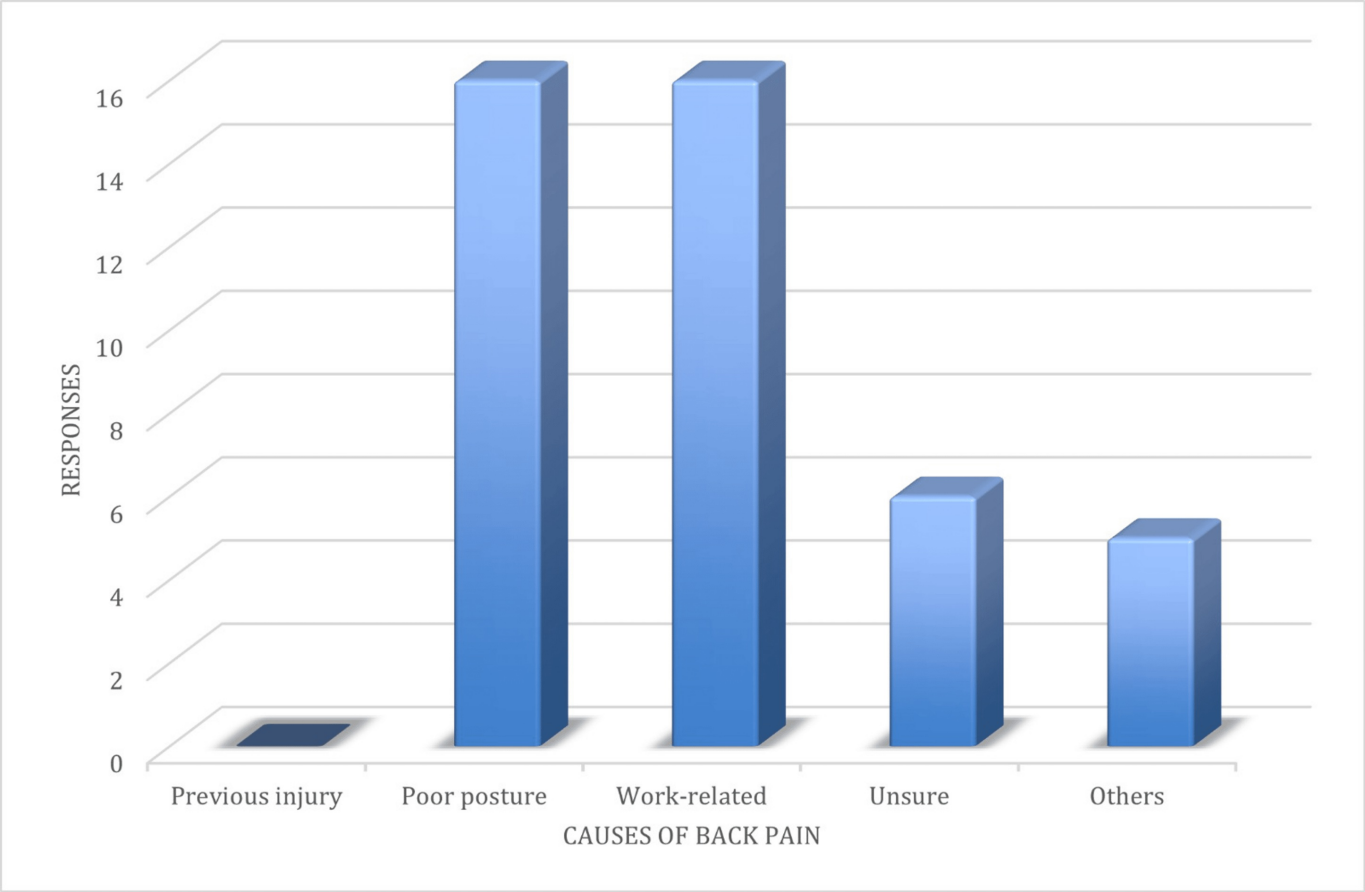
Subjects’ attribution of primary cause of the back pain.

Additionally, three surgeons (6.4%) who were part of the study reported having a previous injury to the back before they started experiencing pain. Most participants (74.4%; N=46) attributed back pain to poor posture and work-related factors. However, 14% (N=9) of the participants were unsure about the cause of their pain, whereas 11.6% (N=7) attributed their back pain to other reasons (Figure 2).

Among the participants, 40.3% (N=25) experienced fatigue due to neck and back pain. Additionally, 32.3% (N=20) reported a decrease in the quality of life due to pain. Of the surgeons involved in the study, 9.7% (N=6) reported altering their medical practices because of pain. Furthermore, seven participants, accounting for 11.3% (N=7) of the total study participants, indicated that they implemented changes in the operating theatre to alleviate the pain. Furthermore, five participants, constituting approximately 7.94% of the study cohort, reported taking days off due to back and neck pain. Lastly, three participants, comprising 4.8% of the study population, considered early retirement due to the pain they experienced in the neck and back region.

## Discussion

Healthcare professionals have a higher incidence of work-related musculoskeletal disorders than those employed in the manufacturing, construction, and transportation industries combined [10]. Orthopedic surgeons are especially susceptible to the unique job stresses that they endure, such as strenuous posture, prolonged standing, and additional extremes of motion required for surgery. These factors contribute to higher rates of musculoskeletal pain than in other surgical subspecialties [9,11]. In our sample, back and neck pain was primarily attributed to poor posture, consistent with findings from previous studies that posture affects pain [3,9].

This study evaluated the prevalence of back and neck pain among orthopedic surgeons in Riyadh, Saudi Arabia. The research further explored potential general factors that could contribute to these symptoms and their effects on the professional and personal lives of the participants. This study found a prevalence of 74.2% (N=46) and 58.1% (N=36) of back and neck pain, respectively. The distribution of symptoms was equal across training levels, suggesting that the problem was not limited to a particular stage of experience or expertise.

Lucasti et al. highlighted the substantial prevalence of musculoskeletal discomfort among surgeons and interventionists on a global scale, with rates ranging from 35% to 60% [9]. Orthopedic surgeons, in particular, experience a high prevalence of back pain ranging from 15.2-89.5% and neck pain from 2.4-74% [5]. This poses a considerable burden due to its potential impact on productivity, career longevity, and quality of life.

Previous Saudi Arabian research found a notably high prevalence of lower back (74.0%) and neck (58.2%) pain among orthopedic surgeons, consistent with our findings [7]. More recent studies in the eastern region of Saudi Arabia have indicated that lower back (46%) and neck (39.7%) pain are the most common, with the highest prevalence among younger residents [8]. Research in the northwestern region of Saudi Arabia reported back pain in 85.4% and neck pain in 37.5% of orthopedic surgeons [12]. Although the majority of our sample performed two to three operations daily, as in other Saudi studies, the prevalence may be higher in Riyadh owing to its advanced healthcare centers and high service demand [13]. As the country's medical hub, workloads, and associated stressors could exceed those in other regions, potentially elevating musculoskeletal issues. Further investigations may help clarify regional differences.

Our research revealed that only a minority of individuals with back or neck pain sought medical care. Specifically, 34.8% (N=16 individuals) of those with back pain and 33.3% (N=12) of those with neck pain pursued treatment, with only 3.2% (N=2)receiving active treatment for their condition. Notably, those reporting moderate-to-severe pain were more likely to seek care, indicating that higher pain levels prompted action. Interestingly, our findings suggest that approximately 35% (N=22) of the surveyed orthopedic surgeons reported pain levels that could warrant narcotic pain relief, according to some institution-based postoperative pain control guidelines [10,14].

This is alarming, given their expertise as healthcare professionals who are presumably aware of the risks of untreated pain. One possible interpretation is a cultural or professional tendency to downplay or endure pain, perhaps stemming from expectations of resilience or concern about appearing weak [10,11,15].

Study limitations

Our study's cross-sectional design and the random allocation of our questionnaire sample might contribute to selection bias. The modest sample size (N=62) and the response rate could limit the generalizability of our findings. Furthermore, the unequal distribution of participants across specialties may reflect skewed prevalence rates of musculoskeletal conditions. It is essential to acknowledge that this questionnaire is designed solely to capture subjective opinions rather than provide evidence-based insights into the etiology of the pathology. 

Addressing this problem requires a focus on improving ergonomics in the workplace. Simple adjustments, like better posture and reducing the time spent in stressful physical positions, could make a big difference. More research is needed to find better ways to prevent and manage these issues so that surgeons can continue to provide high-quality care without compromising their own health.

## Conclusions

This study highlights just how common back and neck pain is among orthopedic surgeons in Riyadh, with 74.2% reporting back pain and 58.1% experiencing neck pain. The findings show that poor posture and the physical demands of the job are major contributors. Age also plays a significant role, particularly for neck pain, with surgeons aged 35-44 and those over 55 being more affected. Additionally, it was clear that the severity of pain directly influences whether surgeons seek medical help. Beyond the pain itself, many surgeons reported that these issues affected their quality of life, leading to fatigue, adjustments in how they work, time off, and even thoughts of early retirement.

## Appendices

**Table 7:** Study questionnaire, as adopted from Lucasti C et al. [9]. PGY = Postgraduate year.

Have you received the consent form document included in the recruitment email?
No
Yes
Do you agree that by submitting this survey you allow for the investigators to use your responses in this study?
Yes
No
What is your age?
35-44
55-65
45-55
Less than 35
65+
What is your sex?
Male
Female
Do you currently use tobacco products, or have you used any tobacco products in the past?
Yes
No, and I never have
No, but I have in the past
How many hours do you exercise per week?
Open answer
What is your height?
60"-66" (152.5 - 167.5 cm)
67"-73" (170 - 185.5 cm)
74"-80" (188 - 203 cm)
What is your weight?
100-149 lbs (45 - 67.5 kg)
150-199 lbs (68 - 90 kg)
200-249 lbs (90.5 - 113 kg)
250 lbs plus (113.5 kg)
How would you rate your stress level?
Moderate
Severe
Mild
Very severe
What is your current level of training?
Attending
Resident/fellow
For attending what is your subspecialty?
Spine
N/A
Upper extremity
Pediatrics
Trauma
Others
Foot and ankle
Joint arthroplasty
Oncology
For attending, how many years you have been in practice since completing training?
Open answer
For residents, what is current level of training?
N/A
PGY-5
PGY-4
PGY-5
PGY-3
PGY-1
During an average workday; how long do you spend standing?
3-5 hours
6-8 hours
8 hours plus
Less than 3 hours
On average how many operations dor you perform each week?
Open answer
On average how many of these operations take more than 3 hours?
Open answer
On average how many hours do you operate per week?
Open answer
On average how many hours do you sleep per night?
Open answer
How many days during the average week do you experience any level of back pain?
1 to 3 days
Less than one day
No back pain
Daily
4 to 6 days
If you answered yes to the previous question, how severe is your back pain at its worst?
Mild
Moderate
Severe
In what stage of your career did back pain first present? (if applicable)
During residency
N/A
As an attending
Prior to residency
Was there a specific injury that can be associated with the back pain? (if applicable)
No
N/A
Yes (please describe: as open short answer)
What do you believe is the primary cause of the back pain? (if applicable)
Work-related
Unsure
Poor posture
N/A
Other, (open short question)
How many days during the average week do you experience any level of neck pain?
1 to 3 days
Less than one day
No back pain
4 to 6 days
Daily
If you answered yes to the previous question, how severe is your back pain at its worst?
Mild
Severe
N/A
Moderate
When did you first experience neck pain? (if applicable)
Less than 5 years ago
5 to 10 years ago
10 to 20 years ago
N/A
20+ years plus
In what stage of your career did neck pain first present? (if applicable)
As an attending
N/A
During residency
Prior to residency
Was there a specific injury that can be associated with the back pain? (if applicable)
No
N/A
Posture during surgery
Congenital
What do you believe is the primary cause of the neck pain? (if applicable)
Work-related
Poor posture
N/A
Unsure
Previous injury
Have you ever taken days off because of your back/neck pain?
No
Yes
N/A
Have you ever sought medical care for your back/neck pain?
No
Yes
Are you currently receiving treatment for your back/neck pain?
Not currently but I have in the past
No
Yes,(please describe: open short answer)
Have you ever tried instituting any changes to your operating room to compensate for pain? If yes, describe the changes.
Yes. (please describe: open short answer)
No
Has your back /neck pain caused a decrease in your quality of life?
No
Yes
Has your pain caused you fatigue?
Yes
No
Have you changed the way you practice medicine as a result of your back/neck pain? If yes, describe the changes.
No
Yes, (please: open short answer)
Have you considered early retirement because of your back/neck pain?
No
Yes

## References

[1] (2024). Death on the job: the toll of neglect, 2023. Neglect.

[2] AlOmar RS, AlShamlan NA, Alawashiz S, Badawood Y, Ghwoidi BA, Abugad H (2021). Musculoskeletal symptoms and their associated risk factors among Saudi office workers: a cross-sectional study. BMC Musculoskelet Disord.

[3] Atalan A (2020). Is the lockdown important to prevent the COVID-19 pandemic? Effects on psychology, environment and economy-perspective. Ann Med Surg (Lond).

[4] Harris S (2013). Safety culture in healthcare: the $13 billion case. Prof Safety.

[5] Xu AL, Covarrubias OG, Yakkanti RR, Sotsky RB, Aiyer AA (2023). The biomechanical burden of orthopaedic procedures and musculoskeletal injuries sustained by orthopaedic surgeons: a systematic review. JBJS Rev.

[6] Woodman A, Homan M, Niaz A, Al-Jamea L, Akhtar M, Sager M (2020). Low back pain among healthcare personnel in Saudi Arabia: a systematic review. Ibnosina J Med Biomed Sci.

[7] Al-Mohrej OA, Elshaer AK, Al-Dakhil SS, Sayed AI, Aljohar S, AlFattani AA, Alhussainan TS (2020). Work-related musculoskeletal disorders among Saudi orthopedic surgeons: a cross-sectional study. Bone Jt Open.

[8] Al Mulhim FA, AlSaif HE, Alatiyah MH, Alrashed MH, Balghunaim AA, Almajed AS (2023). The prevalence of musculoskeletal pain (MSP) among orthopedic surgeons and residents in Saudi Arabia's eastern area. Cureus.

[9] Lucasti C, Maraschiello M, Slowinski J, Kowalski J (2022). Prevalence of back and neck pain in orthopaedic surgeons in western New York. J Am Acad Orthop Surg Glob Res Rev.

[10] Larson DR, Crowson CS, Devick KL, Lewallen DG, Berry DJ, Maradit Kremers H (2021). Immortal time bias in the analysis of time-to-event data in orthopedics. J Arthroplasty.

[11] Bruin MK, Brandt DC, Körner FJ (1976). A climate room for precise regulation of temperature and humidity in connection with a temperature-regulated nest site for wood ants. Oecologia.

[12] Aljohani LZ, Batayyib SS, Ghabban KM, Koshok MY, Alshammari AN (2019). Prevalence of musculoskeletal pain among orthopedic surgeons in north west region of Saudi Arabia: a cross-sectional study. J Orthop Res Physiother.

[13] Alnassar A, Aljerian N, Alhosaini A (2022). Trends of referrals throughout the kingdom, a retrospective analysis of the Saudi medical appointments and referrals centre registry, Saudi Arabia. Int J Innov Res Med Sci.

[14] Besch G, Liu N, Samain E (2011). Occurrence of and risk factors for electroencephalogram burst suppression during propofol-remifentanil anaesthesia. Br J Anaesth.

[15] Epstein S, Sparer EH, Tran BN, Ruan QZ, Dennerlein JT, Singhal D, Lee BT (2018). Prevalence of work-related musculoskeletal disorders among surgeons and interventionalists: a systematic review and meta-analysis. JAMA Surg.

